# The late positive potential is associated with serial dependence effects in facial identity

**DOI:** 10.1038/s41598-026-47266-3

**Published:** 2026-04-01

**Authors:** Anette Lidström, Inês Bramão

**Affiliations:** Department of Psychology, Lund University, Allhelgona kyrkogata 16A, Lund, 223 50 Sweden

**Keywords:** Serial dependence, Facial identity, Segregation, Integration, Electroencephalography, Event-related potentials, Neuroscience, Psychology, Psychology

## Abstract

The visual system can exploit knowledge about temporal contexts in two ways. Attractive serial dependence (SD) can facilitate stability by biasing current stimulus judgments toward recent stimulus history, while repulsive SD can promote change detection by biasing current judgments away from recent stimulus history. This study investigated the co-occurrence of repulsive and attractive SD across facial identity similarity using electroencephalography. Participants performed a matching task with morphed facial identities at varying visibility levels. Results revealed repulsive SD effects for similar faces and attractive SD effects for dissimilar faces, regardless of visibility. Event-related potential analysis showed no significant effects of facial similarity on the N170 and N250, while the Late Positive Potential (LPP) was amplified for dissimilar faces and attenuated for similar faces. Regression analysis revealed a statistically significant relationship between the LPP and attractive, but not repulsive, SD effects. Topographical analysis indicated distinct distributions for repulsive and attractive SD effects within the LPP time-window. These findings suggest that the LPP, possibly reflecting attention-dependent comparison processes, is associated with attractive SD in facial identity. Overall, repulsive and attractive SD effects may rely on partially distinct neural generators, indicating that segregation and integration processes in SD are not due to a common underlying mechanism.

**Supplementary Information:**

The online version contains supplementary material available at 10.1038/s41598-026-47266-3.

Visual processing is sensitive to temporal context. The incoming stream of sensory input is partially redundant because the current input often resembles the previous one. The visual system can exploit this temporal redundancy to enhance processing efficiency in two ways. First, it can adapt to changes between input stimuli, thereby discriminating sensory input over time^1^. Second, it can facilitate continuity and stability by integrating recent stimulus history with the current visual input^2^. Both ways may result in serial dependence (SD) in stimulus judgments, which can be either repulsive or attractive. The consequence of adaptation is reflected in repulsive SD effects, where the judgment of a subsequent stimulus is biased away from a previous stimulus^3^. In contrast, the consequence of integration is reflected in attractive SD effects, where the judgment of a current stimulus is biased toward a previous stimulus^4,5^. Such SD effects have been observed across various stimulus domains, such as orientation, position, color, and faces, suggesting they may be a universal property of visual cognition^5^.

The co-occurrence of attractive and repulsive SD effects has been reported most consistently in the orientation domain, and less consistently for color, and position as a result of time, task, and stimulus similarity^5–13^. In the context of stimulus similarity, the observed pattern typically shows attractive SD effects between more similar stimuli, which reverse to a repulsive effect as the similarity between consecutive stimuli decreases^5^. However, it remains unclear whether these two opposing effects also co-occur due to stimulus similarity for more complex stimuli, such as facial identity, or what neural mechanism(s) underlie the pattern of such effects. In this study, the high temporal resolution of electroencephalography (EEG) and event-related potentials (ERPs) was used to investigate the co-occurrence of repulsive and attractive SD in facial identity judgments.

One important goal of the visual system is to stabilize visual experiences over time, especially when sensory input is noisy or incomplete^14^. Attractive SD in facial identity is thought to reflect a coding mechanism that stabilizes face perception and facial representations in memory by integrating faces over short periods of time^15–17^. Consistent with this notion, the attractive bias toward the previous face is typically greatest when the previous and current face fall within the range of highly physically similar faces along a “morphed continuum”^5,16^. The tuning of the attractive bias to similar faces is thought to operate on high-level, abstract representations of identity rather than solely on low-level visual features^17^. This is demonstrated by findings that attractive SD effects persist across changes in low-level features such as a face’s viewpoint (for example, a three-quarter view following a frontal view), but not for rotations around the roll axis or for face inversions that disrupt face recognition^15,16,18^.

Individual differences in how selectively this bias occurs for highly similar faces are associated with general face recognition abilities. Individuals with better face recognition tend to have narrower, more selective tuning for highly similar faces as compared to those with poor face recognition abilities^16^. Furthermore, autistic traits such as a more detail-oriented processing style are associated with attractive SD effects across a wider range of facial similarities, possibly reflecting an impairment in holistic face processing that may reduce the ability to discriminate between facial identities^19,20^. Selective tuning to highly similar faces makes attractive SD a bias that benefits face recognition if the visual system integrates faces over time only when they are likely to come from the same stable facial identity, which prevents unrelated facial identities from being integrated.

Ensuring that the brain remains responsive to changes in the visual environment by discriminating unrelated sensory input over time fulfills another important goal of perception^3^. In the context of facial identity, previous studies have failed to detect the co-occurrence of repulsive and attractive SD effects due to stimulus similarity but instead show an attractive SD effect that is absent or reduced as the similarity between facial identities decreases^5^. Nevertheless, the co-occurrence of SD effects for faces has been reported, with attractive SD effects observed for gender and repulsive SD effects for expression judgments, demonstrating that both processes can operate simultaneously on the same stimulus, depending on the stability of the feature being judged^21^. However, judging whether a face is male or female, or happy or sad, requires only broad perceptual categorization as compared to the detailed analysis required to identify the subtle and unique differences between faces involved in identity discrimination^22^.

In the present study, we investigated the co-occurrence of repulsive and attractive SD across facial identity similarity. Because previous findings^16,17^ suggest a relationship between face recognition, facial similarity, and SD effects, we manipulated facial visibility, as it is known to affect face recognition^23–25^. We used a matching task based on a morphed continuum, in which two consecutive facial identities were overlaid with a scene image at two different transparency levels on each trial. To determine the effect of facial similarity on attractive and repulsive SD effects, we analyzed the data according to three distance ranges between the two consecutive faces. Based on previous findings from the orientation domain^5^, we expected to observe repulsive SD effects for dissimilar faces and attractive SD effects for similar faces. However, if face recognition modulates the association between facial similarity and SD^16,17^, faces that are less, as compared to those that are more visible, may result in SD effects across a wider range of stimulus distances, which may affect the co-occurrence of attractive and repulsive SD effects. EEG was recorded throughout the experiment, permitting ERP analyses to investigate the neural effects of stimulus similarity and visibility on various stages of face processing to examine the contribution of the mechanism(s) underlying these stages to SD effects.

We focused on ERP components sensitive to face discrimination, as identified in previous studies. One such component is the N170, an early marker of face processing, which shows a negative deflection in the ERP waveform approximately 170 ms after the presentation of a face and is especially large and pronounced over the parieto-occipital scalp areas, particularly the right hemisphere^26,27^. The N170 component is thought to reflect early structural encoding of faces, including features such as the eyes, mouth, and overall facial configuration^28^. An attenuated N170 response to a face preceded by the same, as compared to a different face or non-facial stimuli, is commonly observed, which has led to the conclusion that the N170 is sensitive to individual face discrimination^26,29–32^. However, some evidence questions this notion, suggesting that the N170 reflects facial category rather than identity discrimination^33,34^. The amplitude of the N170 for a previous face predicts attractive SD effects when the face is viewed under concurrent high, as compared to low, working memory (WM) load, such that a smaller N170 response indicates stronger SD effects^35^.

Another early face-related ERP component associated with facial identity discrimination is the N250^34^. The N250 is a negative deflection over parieto-occipital scalp areas in the ERP waveform that occurs approximately 250 ms after the presentation of a face^36^. The N250 is thought to reflect recognition and identity-level processing and to indicate the activation of stored face representations when matching visual input to memory-based identity information^34,36^. An attenuated N250 amplitude to a subsequent facial identity following prolonged exposure to a different identity is thought to indicate that identity-selective neurons have adapted, supporting the notion that facial identity discrimination occurs after structural encoding^34,36^.

In addition to early face-related ERPs, it has been proposed that the late positive potential (LPP, sometimes referred to as late P3), which is associated with context updating, detection of perceptual novelty, and a comparison process dependent on attention^37,38^, is sensitive to facial identity discrimination^39^. The LPP is a positive deflection over the centroparietal scalp area occurring approximately 400 to 600 ms after stimulus presentation and has shown to be more negative for a face preceded by a similar, as compared to a dissimilar face^37,39^. Gao and Wang^39^ found that adaptation to a previous face was accompanied by a suppressed amplitude and prolonged latency of the LPP in response to a subsequent face. Specifically, there was a proportional trend such that the strongest suppression of the LPP occurred when the previous and subsequent face was most similar. As the similarity between faces decreased, the LPP gradually recovered from suppression. These LPP modulations due to identity similarity between the previous and subsequent face suggest that the proportionally suppressed and prolonged LPP activity is sensitive to identity discrimination, occurring at a later stage of face processing^39^.

In the present study, we examined the effects of facial similarity and facial visibility on the N170, N250, and the LPP relative to the onset of the second face. To determine whether there were additional ERPs related to SD facial identity effects beyond the preselected ERPs mentioned above, we also conducted an exploratory cluster-based analysis on the EEG data at three separate time-windows, from the onset of the second face to 1,200 ms after stimulus onset. The behavioral SD effects were regressed on ERP effects due to variations in facial similarity and facial visibility to determine the contribution of the mechanism(s) underlying these ERPs to SD effects.

## Methods

### Participants

Twenty-nine participants, recruited from among psychology students at Lund University and via the Swedish online recruitment website Accindi (www.accindi.se), took part in the experiment. One participant was excluded due to excessively noisy EEG data, so the final sample consisted of twenty-eight participants (21 women and 7 men; age range 20–30 years). All participants reported normal or corrected-to-normal visual acuity and no neurological or psychiatric diagnoses. All participants received a gift voucher upon completion of the experiment.

The study was conducted in accordance with the ethical guidelines of the Swedish Research Council’s Ethics Committee and the European Research Council’s Ethics Committees for research involving human participants. Prior to their participation, all participants gave their written informed consent and were informed about the experimental procedure and their right to withdraw from the study at any time without consequence. As established by Swedish authorities and specified in the Swedish Act concerning the Ethical Review of Research involving Humans (2003:460), the present study does not require specific ethical review by the Swedish Ethical Review Board for the following reasons: (1) it does not involve sensitive personal data, (2) it does not use methods involving physical intervention, (3) it does not use methods that pose a risk of mental or physical harm, and (4) it does not study biological material taken from a living or deceased person that can be traced back to that individual. Additionally, the Ethics Committee at the Department of Psychology, Lund University, has confirmed that the present research protocol follows the research ethics guidelines established by Swedish authorities.

### Material

*Facial stimuli.* The stimuli used for the facial matching task were grayscale male facial morphs generated from three original facial images from the Face Research Lab London Set^40^. A set of 46 facial morphs was created between each of the three original facial images, resulting in a morph wheel comprising 141 facial morphs. The facial images showed neutral expressions and were cropped using an oval aperture to remove the hairline. The morph wheel was created in Webmorph^41^.

**Fig. 1:**
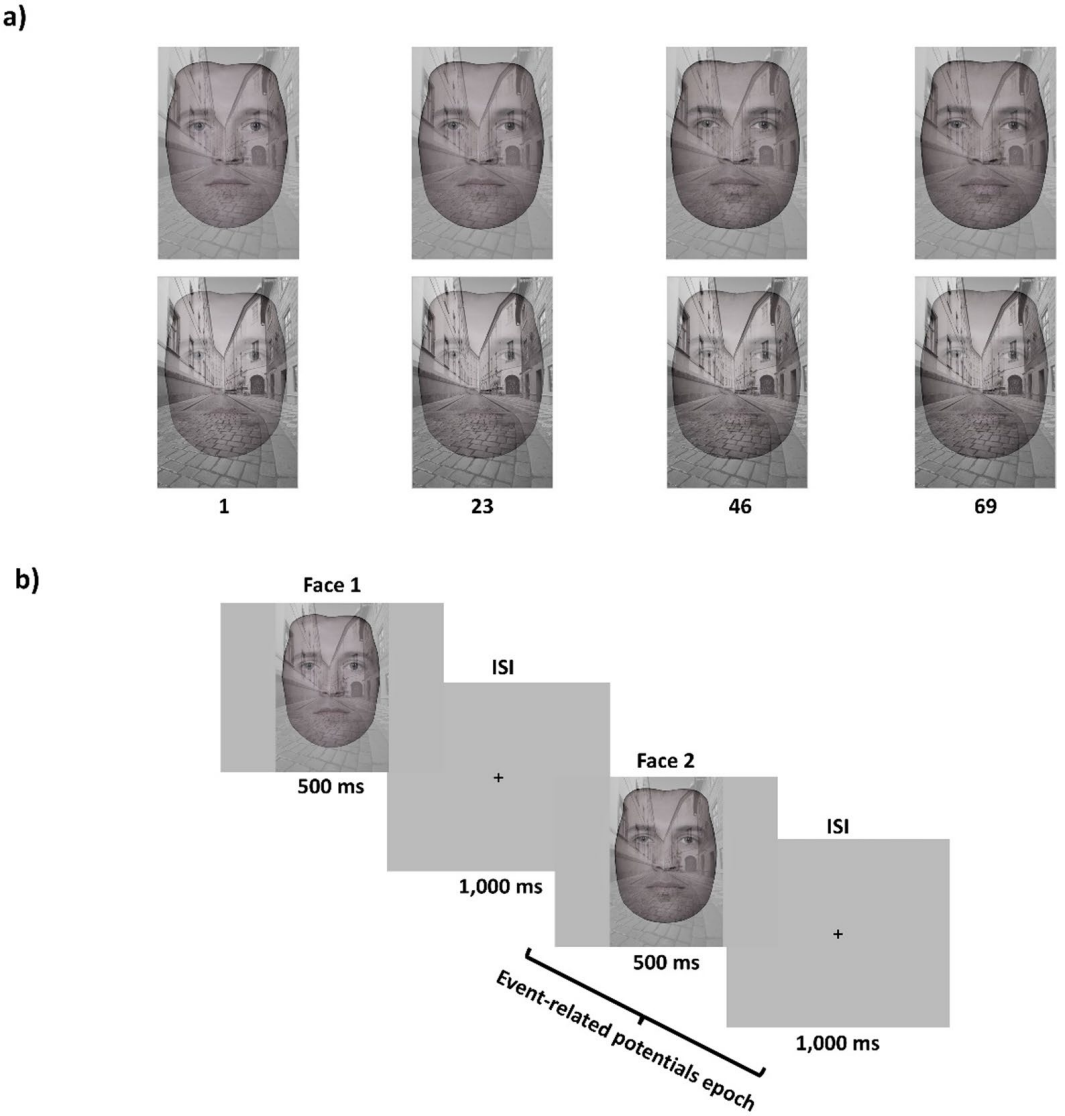
Stimuli material and procedure. (a) Example images from the morph wheel. The top row illustrates facial images at high visibility, and the bottom row illustrates facial images at low visibility. The numerical values indicate the similarity of the faces by the number of morph steps from image 1. (b) Procedure. On each trial, two consecutive facial images at high or low visibility were presented. Face 1 was always an irrelevant inducer face and Face 2 was always the target face to be memorized and reproduced in the self-paced adjustment response. All faces in the figure were selected from the open access Face Research Lab London Set^40^.

Building on the method of Sreenivasan et al.^25^, the morphed facial images were superimposed with an image of a scene showing an empty street, i.e., no people or animals were present in the scenes. The visibility of the faces was manipulated by adjusting the transparency of the superimposed scene image. At low visibility, the transparency of the scene image was 50%, and at high visibility, the transparency of the scene image was 75%. To avoid any effects of stimulus differences other than the identity of the faces and visibility, the same scene image was used for the different facial morphs. Example images from the morph wheel are illustrated in Fig. 1a. All stimuli were presented in the center of the monitor against a light gray background. Figure 1 Stimuli and Procedure.

### Procedure

Each participant was tested individually in the EEG laboratory of the Department of Psychology at Lund University. The experiment comprised eight blocks of twenty-four trials (50% low visibility and 50% high visibility trials). Figure 1b shows an illustration of one experimental trial.

On each trial, two consecutive facial morphs (Face 1 and Face 2) randomly selected from the morph wheel were presented at low or high visibility. The visibility of the facial morphs was always the same for Face 1 and Face 2 on each trial. Both faces were presented for 500 ms each, separated by an interstimulus interval (ISI) of 1,000 ms in which a fixation cross was present. Participants were instructed to look at both Face 1 and Face 2 but informed that Face 2 was always the target face to be memorized, and that Face 1 was irrelevant to the response task. Following Face 2, a fixation cross was present during a 1,000 ms ISI, followed by a grid of 1 or 3 cartoon face(s) presented for 4,000 ms (low/high WM load) before a 1,000 ms ISI, after which a match face randomly selected from the morph wheel was presented. Participants used the left and right arrow keys to adjust the match face until it looked like Face 2. Once satisfied with their selected match face, participants pressed the down arrow key to submit their response. The match face was always presented without the superimposed scene image, and the adjustment task was self-paced. Because the cartoon faces were part of a WM task that is not relevant to the research question of this paper, they will not be discussed further.

Each participant completed 192 trials in one experimental session, totaling 5,376 trials across all participants. Participants were allowed to take breaks between blocks, and the experimental session lasted an average of 60 min. Participants’ EEG was recorded throughout the entire experimental session.

The experimental task was run using MATLAB (The MathWorks, Natick, MA) along with the Psychophysics Toolbox (version 3) extension^42–44^.

### Statistical analyses of behavioral data

Trials in the facial matching task with reaction times of more than 15 s and adjustment errors of more than ± 60 morph steps were excluded from the analysis^15^. As a result, 3% of all trials were excluded. To estimate robust repulsive and attractive SD effects for each participant, we used a nonparametric analysis^12^ conducted within three distance ranges (close/middle/far) between Face 1 and Face 2^45^. The close distance range was within ± 1 to 23 morph steps, the middle distance range was within ± 24 to 46 morph steps, and the far distance range was within ± 47 to 69 morph steps (Fig. 1a). One morph step corresponds to one facial morph between Face 1 and Face 2 in the morph wheel. To make the bins even, data for stimulus distances >= ±70 morph steps were excluded. For each participant, visibility condition and distance range, we computed the median signed adjustment error for trials where the distance between Face 1 and Face 2 was clockwise (+) and subtracted that from the median adjustment error on trials where the distance between Face 1 and Face 2 was counterclockwise (-). This resulted in a bias measure which reflected the systematic deviation of adjustment errors from zero where positive values reflect attractive and negative values reflect repulsive SD effects. We then performed a nonparametric two-way repeated measures ANOVA using aligned rank transform^46^ with visibility (low/high) and distance range (close/middle/far) as within participant factors. Bayes Factor’s (*BF*) were computed to determine the relative support in the data for competing models. Next, the results of each visibility condition and each distance range were visualized and bootstrapped 95% confidence intervals (CIs) were computed by resampling the data for each visibility condition and each distance range for 5,000 iterations.

A repeated measures ANOVA with the same within participant factors as described above along with *BF*s was also performed to determine whether the adjustment errors and so performance in the matching task were affected by visibility and distance range.

All statistical analyses were computed using MATLAB (The MathWorks, Natick, MA) and R (R Core Team, 2023).

### EEG data acquisition and preprocessing

EEG recordings were obtained from each participant throughout the experimental session. EEG data were recorded using a Grael 4 K amplifier (Neuroscan, Compumedics Limited, Australia) at a sampling frequency of 2,048 Hz using 31 Ag/AgCl electrodes arranged according to the extended international 10–20 system. Two electrodes placed over the left and right mastoid served as references. Additional electrodes were used to record the vertical and horizontal electrooculograms (VEOG and HEOG), with the VEOG electrodes placed above and below the left eye and the HEOG electrodes placed at the left and right outer canthi. The impedances of the electrodes were kept below 5 kΩ throughout the experimental session.

The EEG data were preprocessed using FieldTrip^47^ and customized MATLAB scripts. The continuous EEG data were high-passed filtered at 0.1 Hz. Offline, the data were downsampled to 512 Hz and segmented into an epoch ranging from − 200 to 1,200 ms relative to the onset of Face 2 and transformed to a linked-mastoid reference. In addition, bipolar electrooculograms were calculated to detect vertical eye-blinks and horizontal eye movements. The EEG epoch was visually inspected and muscle activity or other artifacts not related to blinks and horizontal eye movements were manually removed. An independent component analysis was performed and components representing oculomotor artifacts and muscle activity distinct from the EEG signal were removed. Any bad channels were interpolated, and the data were again visually inspected to remove trials with residual artifacts.

To assess the effects of distance range and visibility on repulsive and attractive SD effects, ERP analyses were conducted for Face 2. When analyzing the ERPs, the preprocessed EEG data were subjected to low-pass filtering using a Butterworth filter with a cutoff of 30 Hz and baseline corrections were applied at −200 ms relative to the onset of Face 2. Because a linked mastoid reference can negatively affect parieto-occipital ERPs^48^, we re-referenced the EEG data to a common average before computing the ERPs for the N170 and N250 time-windows.

For the final analysis, the ERP data comprised an average of 55 trials for the close distance range (ranging between 37 and 66 trials, 10% average rejected trials), 55 trials for the middle distance range (ranging between 35 and 64 trials, 11% average rejected trials), 56 trials for the far distance range (ranging between 39 and 66 trials, 11% average rejected trials), 81 trials for low visibility (ranging between 55 and 93 trials, 11% average rejected trials), and 82 trials for high visibility (ranging between 58 and 93 trials, 10% average rejected trials) for each participant.

### Statistical analyses of ERPs

The N170, N250, and the LPP were selected for analysis. For the N170 time-window (140 to 200 ms post Face 2 onset), we selected four channels (P4/P8/PO10/O2) over the right parieto-occipital region and four channels (P3/P7/PO9/O1) over the left parieto-occipital region based on previous findings^26,35,39^. The same channels were selected for the N250 within a time-window of 230 to 330 ms post Face 2 onset^34^. For the LPP, four channels were selected over the centroparietal region (Cz/CP1/CP2/Pz) in a time-window of 400 to 600 ms post Face 2 onset, based on previous work^39^. These time-windows and channels were also confirmed by visual inspection of the grand average waveforms.

To determine the statistical significance of the ERP effects obtained, we analyzed differences between distance range and facial visibility using a nonparametric cluster-based permutation test as implemented in FieldTrip^49^. For distance range, the middle distance range was used as the baseline and the cluster-based permutation test was performed on the difference between the middle and close distance ranges, and the difference between the middle and far distance ranges. Differences between high and low visibility were analyzed separately within the close and far distance ranges.

Paired samples *t*-tests were first performed to separately compare distance range and visibility conditions and identify data samples of statistical significance, following the conventional criterion of alpha < 0.05. Adjacent samples were then grouped into clusters, and the *t*-values for each cluster were summed to obtain a cluster-level *t*-value. False discovery correction was conducted by evaluating the cluster-level test statistic with respect to the randomization null distribution of the maximum cluster-level test statistic. This analysis involved randomizing the data between distance ranges and visibility conditions for each participant. A reference distribution of 10,000 random permutations was generated, from which Monte Carlo *p*-values were estimated based on the proportion of the randomization null distribution that exceeded the observed cluster-level maximum test statistic.

To determine whether there were additional ERP effects associated with attractive and repulsive SD effects in facial identity beyond the preselected ERPs, the same nonparametric cluster-based permutation test including all channels was also performed separately in the time-windows from 0 to 400, 400 to 900, and 900 to 1,200 ms during the Face 2 epoch. As a result, statistically significant clusters were identified across the dimensions of time, amplitude and channels. For the statistically significant clusters obtained in the cluster-based analysis, Cohen’ s *d* was computed as a measure of effect size. *BF*s based on paired samples *t*-test were also computed for all ERP comparisons.

The only ERP effects observed from variations in facial visibility were N170 effects. However, regression analyses showed no statistically significant relationship between these effects and SD. For completeness, these results are reported in the Supplementary material (Fig. 1). The following analyses therefore refer to the ERP effects obtained due to variations in distance range, which showed interpretable effects relevant to the research question.

To determine whether the neural mechanisms underlying repulsive and attractive SD effects were shared or distinct between the middle vs. far and middle vs. close distance ranges, a topographical analysis of the ERP effects obtained within the time-window of the LPP was conducted. Amplitude differences due to variations in distance range on a set of representative channels, (Fz/FC1/FC2/FCz), (C3/C4/Cz/CP1/CP2/CP5), and (Pz/P4/O2/P3/O1/Iz/PO9/P7), were vector scaled^50,51^ and then subjected to a non-parametric repeated-measures ANOVA using aligned rank transform. The within-participant factors included distance range (middle to far × middle to close), region (frontocentral × centroparietal × posterior), and hemisphere (left × midline × right). To assess the relative support in the data for competing models, *BF*s were computed.

To determine whether the ERP effects were associated with the attractive and repulsive SD effects obtained, we conducted robust regression analyses using iteratively reweighted least squares in which behavioral difference scores were regressed on ERP amplitude differences due to variations in distance range. To this end, the behavioral SD and ERP effects in the middle were subtracted from those in the far distance range and the behavioral SD and ERP effects in the middle were subtracted from those in the close distance range for each participant.

## Results

### Behavioral results

**Fig. 2:**
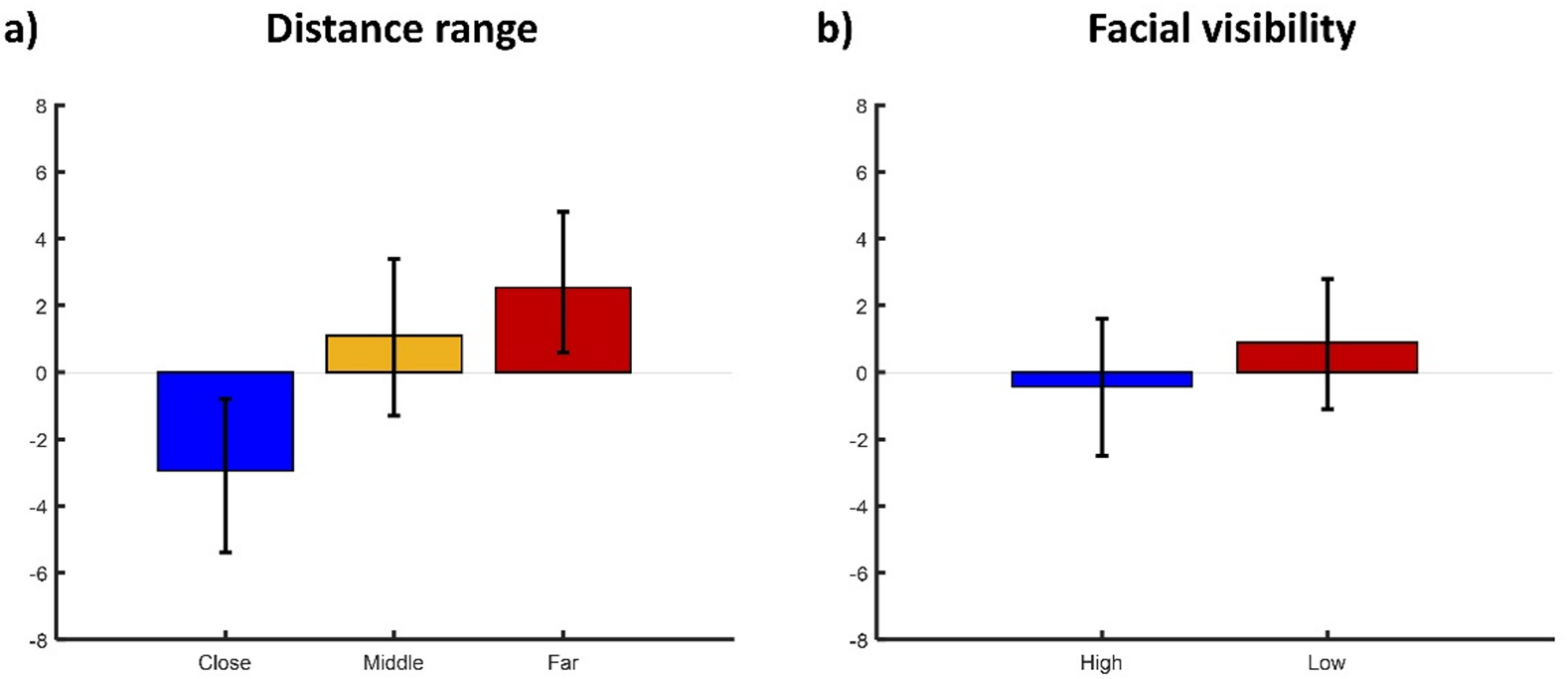
Behavioral results. (a) SD effects for distance range averaged over facial visibility. (b) SD effects for facial visibility averaged over distance range. The error bars represent 95% CIs.

A two-way repeated measures ANOVA showed no statistically significant interaction between visibility and distance range, *F*(2, 162) = 1.67, *p* =.191, *BF*_*10*_ = 1.4. Simple main effects analysis showed a statistically significant effect of distance range, *F*(2, 162) = 5.6, *p* =.004, *BF*_*10*_ = 12.5, but not of visibility, *F*(1, 162) = 0.36, *p* =.549, *BF*_*10*_ = 0.3, on SD effects (Fig. 2a and b). Averaged over visibility, statistically significant repulsive SD effects were obtained within the close distance range, *M* = −2.95, 95% CI [−5.4, −0.8], while statistically significant attractive SD effects were obtained within the far distance range, *M* = 2.54, 95% CI [0.6, 4.8]. No statistically significant repulsive or attractive SD effects were obtained within the middle distance range, *M* = 1.1, 95% CI [−1.1, 3.4] (Fig. 1a). Figure 2 Behavioral Results.

A two-way repeated measures ANOVA showed no statistically significant interaction or main effects of visibility and distance range on adjustment errors: visibility × distance range, *F*(2, 162) = 0.13, *p* =.169, *BF*_*10*_ = 1.97, visibility, *F*(1, 162) = 1.25, *p* =.266, *BF*_*10*_ = 0.04, distance range, *F*(2, 162) = 0.06, *p* =.943, *BF*_*10*_ = 0.01.

Overall, these results suggest that repulsive SD effects were more prominent within the close distance range, while attractive SD effects were more prominent within the far distance range, regardless of facial visibility. The results further suggest that the effects of variations in facial visibility and distance range on adjustment errors, and so on performance in the matching task, were comparable.

### ERP results

**Fig. 3:**
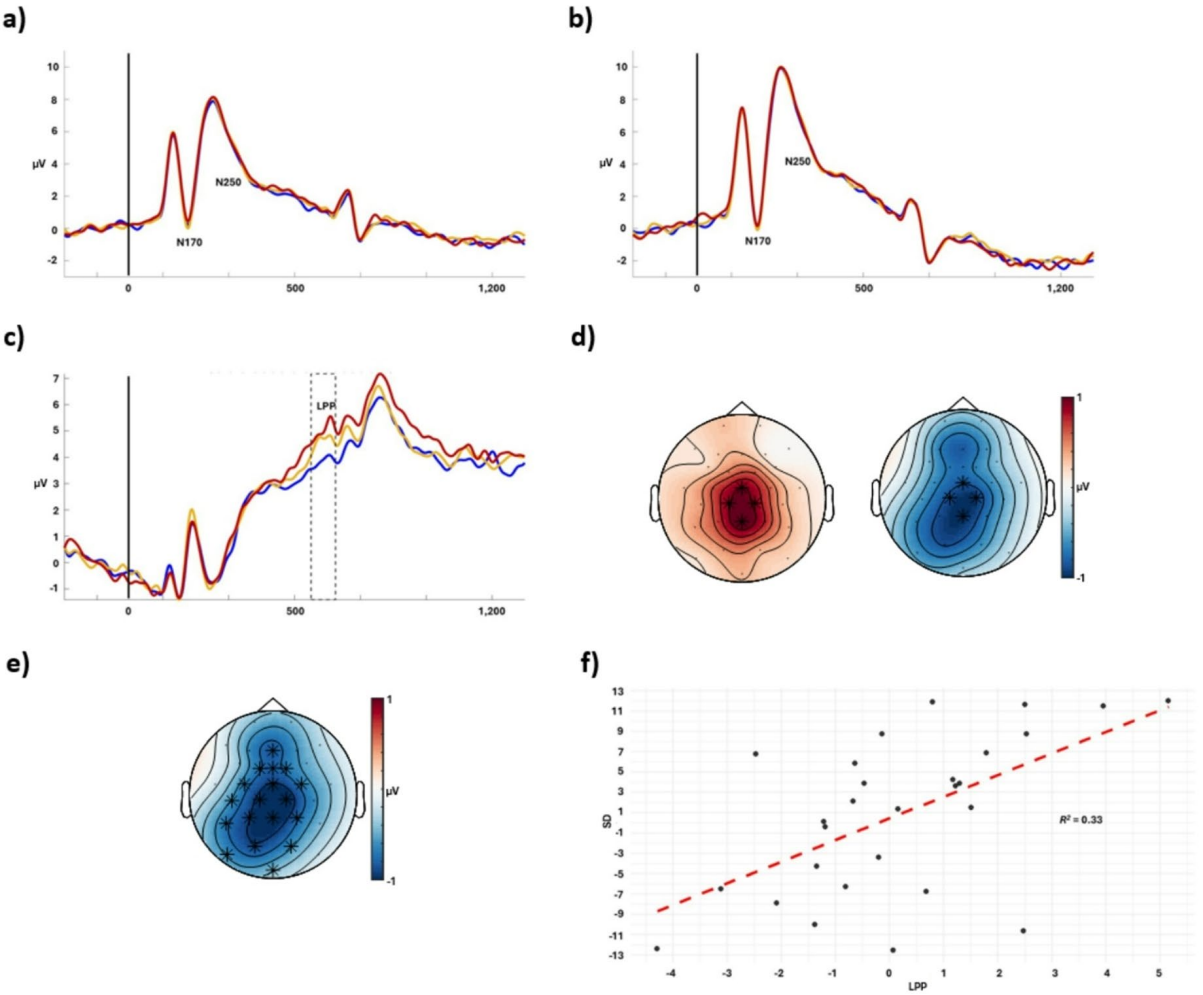
ERP results. (a) Left N170 and N250. (b) Right N170 and N250. (c) LPP and the statistically significant cluster between the middle and close distance range obtained from the exploratory cluster analysis (dashed lines). Blue line = close distance range, yellow line = middle distance range, red line = far distance range. (d) Topographic distributions of the difference between the middle and far distance range (left) and the middle and close distance range (right) for the LPP. (e) Topographic distribution of the difference between the middle and close distance range corresponding to the cluster obtained in the exploratory cluster analysis. (f) Regression plot between the LPP and behavioral difference scores between the middle and far distance ranges. Positive values means that the attractive SD effect and the LPP increased from the middle to the far distance range.

Figure 3 illustrates the grand average ERP waveforms for the N170, N250 (a and b) and the LPP (c). During the N170 and the N250 time-windows, no clusters were obtained over the right or left parieto-occipital region, Fig. 3a and b, and Fig. 1, Supplementary material. In the LPP time-window, we observed an increased positive-going amplitude over the centroparietal channels for the far as compared to the middle distance range, *p* =.014, *d* = 0.9, *BF*_*10*_ = 10.6, and an increased negative-going amplitude over the same region for the close as compared to the middle distance range, *p* =.029, *d* = 1, *BF*_*10*_ = 4.6 (Fig. 3c and d). As illustrated in Fig. 3f, the LPP amplitude attributable to the difference between the middle and far distance range was a significant predictor of the behavioral difference scores between these difference ranges, *F*(27, 1) = 12.8, *p* =.001, *R*^*2*^ = 0.33. No significant relationship was obtained between the LPP amplitude difference and the behavioral difference scores between the middle and close distance ranges, *F*(27, 1) = 0.34, *p* =.565, *R*^*2*^ = 0.01.

To determine whether the neural mechanisms underlying repulsive and attractive SD effects were shared or distinct between the middle vs. far and middle vs. close distance ranges, a topographical analysis of the ERP effects within the LPP time-window was conducted. The topographical analysis revealed a just significant two-way interaction between distance range and region *F*(2, 990) = 2.63, *p* =.05, *BF*_*10*_ = 4.84. Between the middle and close distance range, the ERP effect was significantly stronger at posterior as compared with frontocentral regions, *p* =.024. The ERP effect between the middle and far distance range showed a more widespread distribution that was comparable over the three regions. No additional interactions were obtained: distance range × hemisphere, *p* =.954, *BF*_*10*_ = 1.2, region × hemisphere, *p* =.982, *BF*_*10*_ = 0.9, distance range × region × hemisphere, *p* =.993, *BF*_*10*_ = 0.

Overall, these results indicate an association between the LPP and the attractive, but not the repulsive SD effects and that the topographic distributions between the middle vs. far and the middle vs. close distance ranges were not the same.

The exploratory cluster-based analysis identified a statistically significant LPP-like effect between the middle and close distance ranges within an approximately 550 to 650 ms time-window relative to the onset of Face 2, *p* =.018, *d* = 2.3, *BF*_*10*_ = 14.9 (Fig. 3c and e). Similar to what was previously observed, the amplitude difference between the middle and close distance ranges for the LPP-like effect identified in the exploratory cluster analysis was not a significant predictor of behavioral difference scores between these distance ranges, *F*(1,27) = 0.35, *p* =.558, *R*^*2*^ = 0.01. No other statistically significant ERP clusters were obtained within the predetermined time-windows. Figure 3 ERP Results.

Overall, the results suggest that facial similarity, rather than facial visibility, had a significant impact on SD effects. Repulsive effects were more prominent for similar faces, while attractive effects were more prominent for dissimilar faces. Furthermore, the ERP results confirm that the LPP is sensitive to facial similarity and suggest that the mechanism(s) underlying the LPP are associated with attractive, but not repulsive, SD effects in facial identity.

## Discussion

In the present study, we analyzed ERPs and psychophysical data to investigate the co-occurrence of attractive and repulsive SD effects in facial identity and the neural mechanisms underlying such effects. Repulsive SD effects were obtained for similar faces, while attractive SD effects were obtained for dissimilar faces, suggesting that discrimination occurred between similar faces and integration occurred between dissimilar faces. Additionally, we demonstrated that attractive, but not repulsive, SD effects are associated with the LPP. The present work extends previous findings on the co-occurrence of repulsive and attractive SD effects by showing that the mechanism(s) underlying the LPP, which occurs at a later stage of stimulus processing, predicts SD effects in facial identity.

The present study provides novel insights into the neural mechanisms underlying SD effects in facial identity. The LPP is considered sensitive to facial similarity and identity discrimination^39^. Specifically, a more attenuated LPP amplitude has been observed for a subsequent face that closely resembles a previous face, as compared to when the consecutive faces were more different^39^. We corroborated the similarity-specific sensitivity of the LPP by comparing the effects of similar and dissimilar faces and tested its association with SD by regressing the behavioral SD effects on the LPP effects. Consistent with previous work^37,39^, we observed an attenuated centroparietal LPP in relation to Face 2 when it was preceded by a similar face, and an amplified LPP when Face 2 was preceded by a dissimilar face. These findings were partially associated with the behavioral SD effects: attractive SD effects between more dissimilar faces were associated with a more positive LPP amplitude, while no significant association between repulsive SD effects and the LPP was obtained.

The LPP is thought to reflect context updating, detection of perceptual novelty, and a comparison process dependent on attention^37,38^. Consecutive stimulus presentations can trigger comparison judgments^52^. One possible explanation for our findings and the LPP amplitude differences related to the similarity between Face 1 and Face 2 is that more attentional resources were directed toward Face 2 when the faces were more similar, as compared to when they were dissimilar. ERP amplitude with a similar topography within an approximately 400 to 600 ms time-window post stimulus onset is considered sensitive to the amount of attentional resources engaged in the experimental task^38^. More demanding tasks, as compared to less demanding ones, elicit a smaller ERP amplitude within this time-window, possibly reflecting attentional resources being allocated to task performance^38^. This is consistent with the notion that two similar stimuli presented within the same trial may promote comparison to actively separate them^53^, which may require more attentional resources, as reflected in the attenuated LPP observed for more similar faces.

Previous findings suggest that greater attention to faces results in stronger adaptation effects^54^, which is consistent with the adaptation effects and attenuated LPP for more similar faces reported by Gao and Wang^39^. However, we found no significant association between the repulsive SD effects for more similar faces and the LPP, which may indicate that repulsive SD effects and facial identity adaptation effects due to prolonged exposure to a previous face are two distinct forms of adaptation. However, Gao and Wang^39^ did not analyze the relationship between the behavioral effect and the LPP effect, which somewhat limits their conclusion that the LPP serves as a neural correlate of facial identity adaptation effects. Nevertheless, we advise interpreting the absence of significant relationships between the repulsive SD effects and the obtained ERPs with caution. These results may simply indicate that the relationship between the neural response and the repulsive SD effects is less clear and may reflect a different underlying mechanism or a more complex relationship not captured in the present study.

However, the topographic distributions of the LPP differed between the repulsive and attractive SD effects. This suggests that, although both effects occur within the general temporal window of the LPP, they may rely on partially distinct neural generators or functional processes. In other words, the LPP may serve different computational roles for attractive versus repulsive SD effects, supporting that the mechanisms driving the two types of SD effects may not be simple mirror opposites. Nevertheless, our results suggest that the attractive SD effects obtained for dissimilar faces may be partially due to insufficient attention, as indicated by a more positive LPP, which impaired face discrimination and instead facilitated integration. Interestingly, attractive SD effects are known to occur primarily between similar faces and are thought to rely heavily on attention^5,15^. Our findings may therefore indicate that insufficient attention may shift the effect, resulting in attractive SD also occurring between dissimilar faces.

Attention is a cognitive function that interacts closely with WM, and information maintained in WM is susceptible to interference from concurrently maintained information^53,55,56^. Consistent with our results, attractive SD effects have been found for low-level stimuli such as highly dissimilar oriented bars, while repulsive SD effects were observed for more similar oriented bars concurrently maintained in WM. Furthermore, heightened attention to the stimuli appeared to strengthen the repulsive SD effects^53^. Previous work also shows that mere attention to a preceding face processed during concurrent WM load can facilitate unintentional maintenance of this face in WM, reflected in the EEG signal as a negative slow wave, resulting in attractive SD effects^35^. Given that the time-window of the LPP effect predictive of the attractive SD effects in this study occurred at a later stage of stimulus processing, it is likely that these effects are also related to WM mechanisms.

An alternative explanation is that the ERP effects (observed in both the planned and exploratory analyses) for similar faces may reflect reduced surprise. Surprise can be considered a measure of the difference between sensory input and predictions and is greater for unexpected sensory input or when the discrepancy between sensory input and predictions is larger^57^. However, surprise is generally associated with the mismatch negativity ERP, which occurs relatively early in perceptual processing (150 to 250 ms post-stimulus onset) and is more negative for unexpected or deviant events^57^. Given that the ERP effects between the middle and close distance ranges occurred much later and were more negative for similar faces, which can be considered less deviant, it is unlikely that these effects reflect reduced surprise.

The ERP effects obtained between the middle and close distance ranges could also be consistent with increased WM demands, indicating that similar, as compared to dissimilar faces, required more WM resources^58^. Alternatively, these ERP effects may correspond to recollection processes, such that when the faces were more similar, Face 2 was correctly identified as a new stimulus, which typically elicits a more attenuated ERP within this time-window than correct recollection^59^. Nevertheless, we found no evidence for a significant relationship between the repulsive SD effects obtained for more similar faces and these ERP effects, possibly indicating that the repulsive SD effects were not due to WM demands or recollection processes.

We did not find any modulation of the N170 and N250 ERPs due to variations in facial similarity, suggesting that early face processing did not contribute to the SD effects obtained. The amplitude of the N170 has been shown to predict attractive SD effects^35^. However, this association between the N170 and attractive SD was observed for a previous face that was viewed under concurrent WM load. The experimental task in the present study did not use concurrent WM load but instead manipulated the visual appearance of the faces, and the analyses were conducted for the second face. In addition, we obtained statistically significant effects of facial similarity, but not of facial visibility, on SD effects in the present study, which may not relate to early face processing.

The N170 have, in some studies, been associated with facial identity discrimination^26,29–32^. Our results are more consistent with studies that suggest that the N170 reflects facial category rather than identity discrimination^33,34^. The absence of significant effects for the early ERPs in the present study may further support the interpretation that the SD effects obtained in the present study should be attributed to WM rather than perceptual phenomena. Investigation into the processing stages of SD effects in facial identity is ongoing, but it is thought that attractive SD effects for various stimuli, including high-level stimuli such as faces, may occur at the levels of perception, WM, and decision-making^5^.

We did not obtain a significant main effect of facial visibility or a significant interaction between facial visibility and stimulus similarity. Previous findings have shown a relationship between stimulus similarity, face recognition, and attractive SD effects, such that individuals with poor face recognition abilities exhibit attractive SD effects across a wider range of stimulus distances^16,20^. In the present study, we manipulated the visibility of all faces by using two different levels of transparency. It is therefore possible that the difference in transparency between the less and more visible faces was insufficient, which equally affected face recognition and modulated the SD effects. This is supported by the analysis of the adjustment errors, which showed no significant main effect of facial visibility, suggesting that performance for the two visibility conditions was comparable. Our results may therefore suggest that attractive SD effects between more dissimilar faces, due to insufficient attention, contribute to poor face recognition.

Previous studies have failed to detect the co-occurrence of repulsive and attractive SD effects due to stimulus similarity for facial identity^5^. The co-occurrence of such effects obtained in the present study may indicate that manipulating the visibility of faces elicits attentional processes not involved in processing clearly visible faces, contributing to the co-occurrence of the effects. Future research may further investigate this by comparing clearly visible and less visible faces. In contrast to our results, the co-occurrence of attractive and repulsive SD effects in the orientation domain typically shows attractive SD effects for more similar stimuli, which can reverse to repulsive SD effects as the similarity between stimuli decreases^5^. Future studies may investigate whether the inconsistent results relate to different levels of processing (perceptual and memory) or to low- versus high-level stimuli (orientation and faces).

In conclusion, our results show that repulsive and attractive SD effects co-occur as a function of facial similarity also for facial identity. Furthermore, attractive, but not repulsive, SD effects in facial identity may be partially due to a comparison process dependent on attention that occurs within the time-window of the LPP at late stages of face processing. However, the role of the LPP in repulsive SD effects remains somewhat ambiguous and requires further investigation, as it cannot be entirely dismissed. Future studies may manipulate visibility across a wide range of levels and use tasks requiring different processing stages to clarify this role. Nevertheless, our findings indicate that the processes underlying segregation to facilitate face discrimination and integration to facilitate the stability of facial representations in SD effects are not due to a common underlying mechanism.

## Supplementary Information

Below is the link to the electronic supplementary material.

Supplementary Material 1

## Data Availability

The datasets generated during the current study are available from the corresponding author on request.
