# Supplementary material for: The late positive potential is associated with serial dependence effects in facial identity
Source: Sci Rep. 2026 Apr 1;16:11222. doi: 10.1038/s41598-026-47266-3 (PMC13046828; doi:10.1038/s41598-026-47266-3)
Supplement: Supplementary file 1 — Supplementary Material 1 [file 41598_2026_47266_MOESM1_ESM.pdf]

Supplementary Material

Figure 1

ERPs for facial visibility within the close and far distance range and N170 / N250 amplitude differences

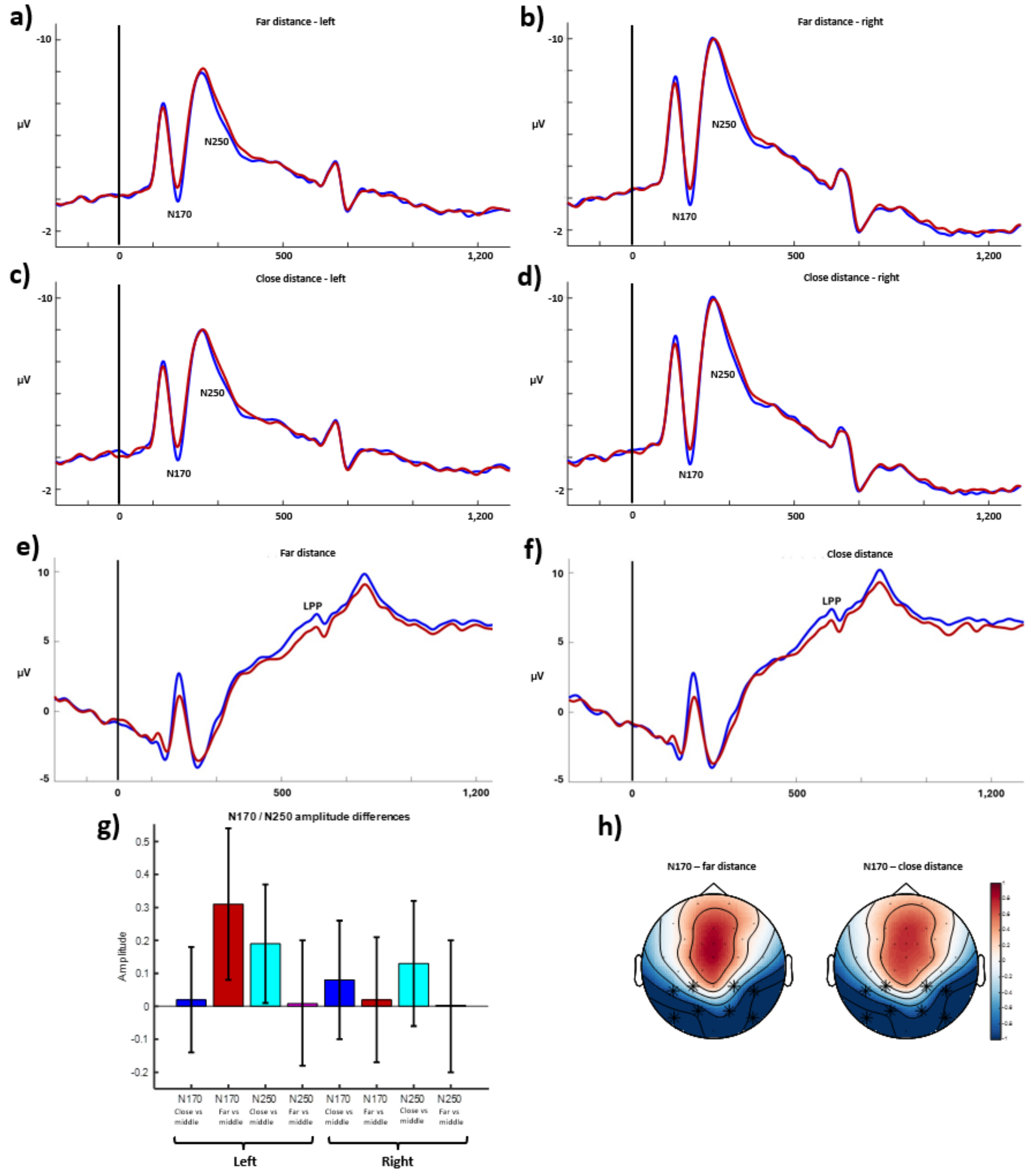

Figure 1. Grand average ERP waveforms for the N170, N250r and the LPP for high and low visibility within the close and far distance ranges and amplitude differences for the N170 and

N250 between the middle to close and middle to far distance ranges. The top row illustrates the left (a) and right (b) N170 and N250 due to variations in facial visibility within the far distance range. The middle row illustrates the left (c) and right (d) N170 and N250 due to variations in facial visibility within the close distance range. The bottom row illustrates the LPP due variations in facial visibility within the far (e) and the close (f) distance range. Low visibility = red, high visibility = blue. Statistically significant clusters for the N170 were obtained between high and low visibility within both the far and the close distance range, showing a significantly more negative N170 for high as compared to low visibility (far distance range: (a and h) left N170, $p = < .001$, $d = 10.2$, (b and h) right N170, $p < .001$, $d = 9.4$, close distance range: (c and h) left N170, $p < .001$, $d = 2.6$, (d and h) right N170, $p = < .001$, $d = 7.5$). Regression analyses revealed no statistical relationship between these N170 effects and SD, all *p*s > .05. (a – f) No statistically significant clusters were obtained between high and low visibility for the N250 and the LPP within either distance range. (g) Amplitude differences for the N170 and N250 within the middle to close and middle to far distance ranges. The error bars represent standard errors.
